# Surgical treatment of multiple large tuberous xanthomas in familial hypercholesterolemia: A case report

**DOI:** 10.1016/j.ijscr.2021.106596

**Published:** 2021-11-12

**Authors:** Pham Thi Viet Dung, Tran Thiet Son, Ta Thi Hong Thuy, Vu Hong Chien, Truong The Duy, Le Van Huy

**Affiliations:** a Department of Plastic Surgery, Hanoi Medical University, No.1 Ton That Tung Street, Hanoi, Viet Nam; b Department of Plastic Reconstructive and Aesthetic Surgery, Bach Mai Hospital, No.78 Giai Phong Street, Hanoi, Viet Nam; c Department of Plastic Reconstructive and Aesthetic Surgery, Hanoi Medical University Hospital, No.1 Ton That Tung Street, Hanoi, Viet Nam

**Keywords:** Tuberous xanthoma, Familial hypercholesterolemia, Case report

## Abstract

**Introduction and importance:**

Xanthomas are a rare condition with the appearance of exogenous masses on the body, and it is common in patients with familial hypercholesterolemia (FH). For multiple large xanthomas, surgical excision is optimal to improve the patient's quality of life.

**Case presentation:**

A 34-year-old male patient presented with multiple large tuberous xanthomas related to FH. There were 15 masses in different body parts, including the dorsum of the hands, elbows, buttocks, feet, and Achille's tendon. The largest masses in the buttocks measured 8 × 8 × 5 cm. Surgical removal of 13 masses was carried out in combination with medical treatment. The skin incision was oval around the circumference of masses with the longitudinal axis parallel to the Langer's line. Skin defects were closed directly or dissected on both sides of the incision to reduce tension. Wound healing was normal. After 1.5 months, there was no recurrence of xanthomas.

**Clinical discussion:**

Surgical treatment easily removes the entire tuberous xanthomas. The healing process is completely normal. Resection should be indicated for tuberous xanthomas that cause negative functional and aesthetic effects. Besides, lipid-lowering therapy is necessary to prevent tuberous xanthomas recurrence as well as premature coronary artery diseases.

**Conclusion:**

Surgical treatment of patients with multiple large tuberous xanthomas related to familial hypercholesterolemia was performed safely and successfully. After 1.5 months of follow-up, the wound healed well and no recurrence of xanthomas was detected. We recommend that a further study is needed to investigate post-treatment recurrence for multiple large xanthomas.

## Highlights

• Surgical treatment for multiple large tuberous xanthomas
• A 34-year-old male patient with masses in different locations of the body
• No recurrence of xanthomas after 1.5 months follow-up

## 1 Introduction

Xanthomas are the appearance of exogenous masses at different locations on the surface of the body. There are many causes of xanthomas, but most often present with severe conditions in patients with familial hypercholesterolemia (FH). Xanthomas are not tumors but a group of foam cells; they are formed by excess free and esterified cholesterol circulating in transvascular plasma deposited in connective tissue, often in the skin or tendons [1], [2]. The masses usually appear in areas of the body exposed to mechanical stress, such as the knuckles, elbows, knees, feet, ankles, and buttocks [7]. The diagnosis of xanthomas associated with FH is often based on the Simon Broome Register or the Dutch Lipid Clinic Network criteria [3], [4]. The manifestations of xanthomas depend on the duration and severity of hypercholesterolemia [5]. One of the most commonly observed xanthomas in patients with FH is tuberous xanthomas, characterized as a conspicuously raised mass on the skin surface [6]. Tuberous xanthomas are unsightly because of their conspicuous location, usually asymptomatic unless they are large enough to cause pain and difficulty in movement. Small tuberous xanthomas and tendon xanthomas may regress with medicament or plasma exchange (LDL-C apheresis) after several months to 12 months [7], [8], [9]. Surgical excision is indicated for patients with large tuberous xanthomas large without regression after lipid-lowering therapy [7], [10]. This paper aims to present a successful surgical treatment for patients with multiple large tuberous xanthomas related to FH. This report has been written in accordance with SCARE guidelines criteria [11].

## 2 Case presentation

**Fig. 1:**
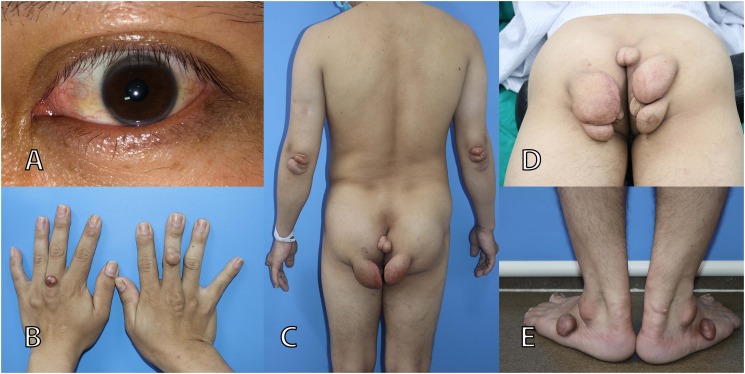
Shows the status of the patient on admission. (A) Arcus cornealis. (B, C, D, E) Tuberous xanthomas in various locations.

A 34-year-old male patient presented to our hospital with multiple yellowish elevated masses in different locations of the body, including the dorsum of the hands, elbows, buttocks and feet. In addition, the patient also had bilateral arcus cornealis and Achille tendon masses (Fig. 1). The total number of masses was 15, and the size of masses varied from 1 × 1 × 1 cm to 8 × 8 × 5 cm. The masses were initially asymptomatic; they appeared 10 years ago, and then they increased progressively in size. The patient complained of discomfort and difficulty dressing and sitting due to large masses in the buttocks. In addition, patients also reported difficulty wearing sandals due to the masses in the feet. The patient's hygiene was affected, and especially the masses made the patient feel inferior when in contact with others. Even the patient decided to divorce because of psychological influences related to abnormalities in the body. The results of the chest X-ray, abdominal ultrasound, electrocardiogram and echocardiogram of the patient were normal. The patient's family medical history was not taken because the patient did not wish to disclose it. The low-density lipoprotein cholesterol (LDL-C) level of the patient was 10.04 mmol/L (reference value, <2.6 mmol/L). Based on a high level of LDL-C, arcus cornealis and presence of xanthomas, the patient was diagnosed with Familial hypercholesterolemia (FH) based on the Simon Broome Criteria.

**Fig. 2:**
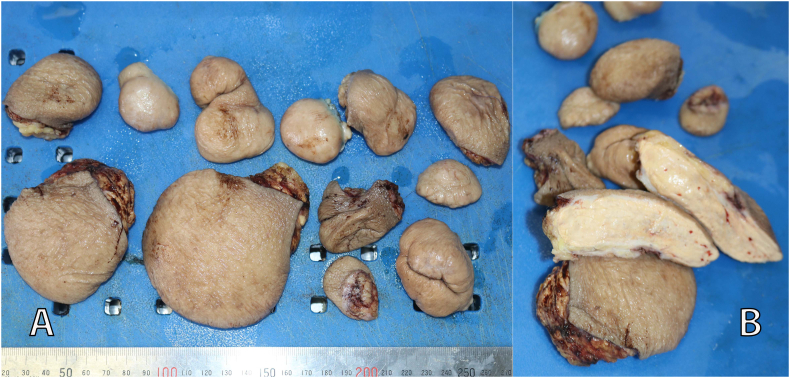
(A) Resected xanthomas. (B) Macroscopic characteristics of xanthomas.

**Fig. 3:**
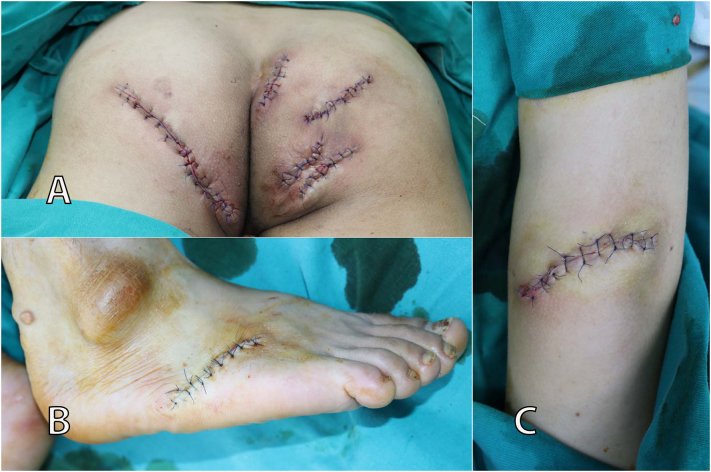
Suture lines parallel to the Langer's line. (A) Buttocks. (B) Foot. (C) Elbow.

**Fig. 4:**
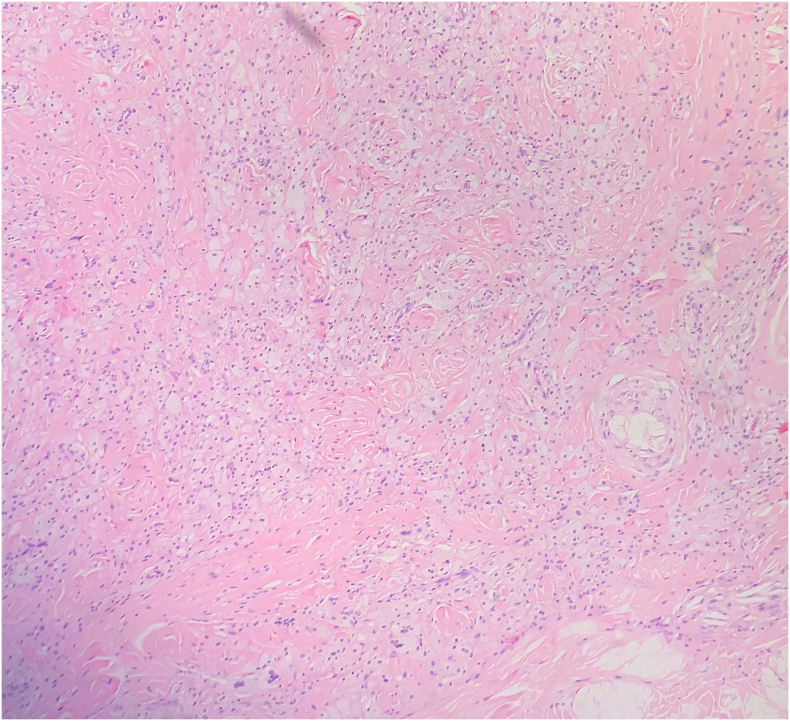
Infiltration of foam cells (vacuolated macrophages) filled with lipid droplets.

**Fig. 5:**
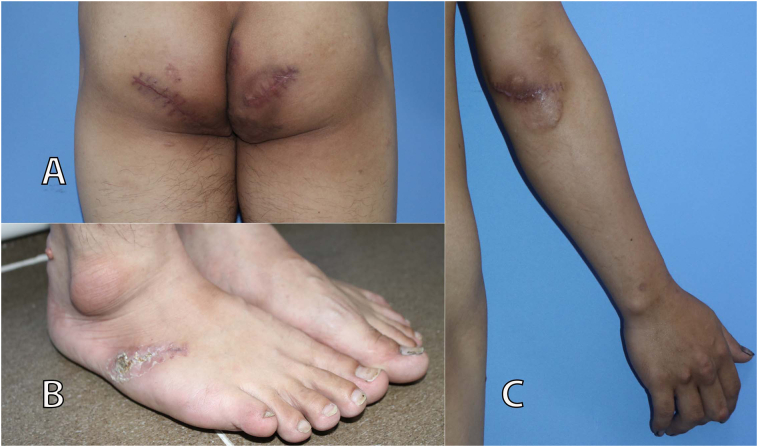
No recurrence was observed after 1.5 months. (A) Buttocks. (B) Foot. (C) Elbow.

After receiving the consultation, the patient agreed to be admitted to the hospital for surgery to remove the masses. Lipid-lowering therapy was immediately administered for the patient with Atorvastatin 40 mg/day. After 4 days of hospitalization, the patient then underwent surgery to remove the masses. The resected masses had a surface of normal skin. The core of the masses looked yellowish-colored uniform, relatively solid, without necrosis, and localized in the subcutaneous layer without invading the muscle or joint capsule (Fig. 2). The skin incision was oval around the circumference of masses with the longitudinal axis parallel to the Langer's line. The excision margins were normal skin. After removing masses, all defects were sutured directly (Fig. 3). A total of 13 masses were removed; the remaining 2 masses on the finger were not removed because the patient still had to use the fingers to take care of himself. The remaining 2 masses would be removed during the next surgery. Histopathology showed a typical xanthoma with infiltration of foam cells (Fig. 4). The healing process was normal. All sutures were removed 14 days after surgery. The patient was discharged and continued treatment with atorvastatin 40 mg/day. Gene sequencing test (on the Nextseq, Illumina system) showed homozygous LDL-C receptor gene mutation on chromosome 19. Due to difficult economic conditions, the patient's family members did not undergo gene sequencing to find mutations causing FH. Follow-up at 1.5 months postoperatively, the LDL-C level of the patient was reduced to 8.8 mmol/L and the wounds healed well and no re-appearance of masses was observed (Fig. 5).

## 3 Discussion

This is the first case of xanthoma-related FH reported in Vietnam. The correct diagnosis of the disease helps in successfully removing masses with good results after 1.5 months of follow-up. This is a valuable experience that can be applied to patients with FH with similar manifestations.

The location of the xanthomas in our patient was similar to that described in previous studies that were present in mechanical stress-exposed parts, such as the knuckles, elbows, knees, feet, ankles and buttocks [12]. Some authors in previous studies proposed theories to explain the reason for the appearance of xanthomas, which are related to an excess of blood lipids, especially in high LDL-C levels [6], [12], [13]. FH is an inherited disorder with an increased LDL-C level, so patients with FH have a high risk of xanthomas [14]. Xanthomas can be divided into several types, the most common of which are tendinous xanthomas and tuberous xanthomas. Our patient's case was diagnosed with tuberous xanthomas with typical manifestations in different locations of the body. Tuberous xanthomas are normally asymptomatic unless they become large and compress adjacent structures [15], leading to pain and mobility difficulties. In our patient's case, the masses became large, entangled, and especially interfered with the patient's daily activities. This was the main reason why the patient came to our hospital, so that this patient was diagnosed as FH.

Lipid-lowering therapy is an option to help with regression in small xanthomas, but moderate to large xanthomas require surgical excision [7], [10]. In a previous study, Zhao et al. reported a case of a patient with multiple large xanthomas [15]. In this case, the removal of xanthomas was also performed and gave good results. Although resection of large xanthomas was reported, the criteria for making that surgical were not clearly addressed. For this case, we believe it is necessary to rely on some of the following criteria for performing xanthomas removal: 1) causing pain; 2) limitation of movement and affect daily activities; 3) affecting psychological and aesthetic. Besides surgical removal of xanthomas, we consider it important to continue with lipid-lowering therapy. Lipid-lowering therapy after xanthomas removal should be used to prevent recurrence and reduce cardiovascular complications [16].

One interesting thing in our case is that we found that xanthomas' longitudinal axis often coincides with Langer's lines. We have searched in previous studies, but we have not found any authors who have mentioned this issue. Perhaps this feature is related to the formation of xanthomas due to excess free and esterified cholesterol circulating in the plasma through the vessels and deposited in the connective tissue [12]. Skin folds are stretched during movement, increasing capillary permeability for LDL-C to pass through. Therefore, xanthomas often form in these regions and develop along the Langer's lines. Therefore, when performing surgery with incision coincides with the Langer's lines tends to heal better and produce less scarring than those that cross them.

## 4 Conclusion

Surgical treatment of patients with multiple large tuberous xanthomas related to familial hypercholesterolemia was performed safely and successfully. During surgery, using an incision that coincides with the Langer's lines can help heal scars better. After 1.5 months of follow-up, the wound healed well and no recurrence of xanthomas was detected. We recommend that a further study is needed to investigate post-treatment recurrence for multiple large xanthomas.

## Abbreviation

FH: familial hypercholesterolemia
LDL-C: low-density lipoprotein cholesterol

## Sources of funding

None.

## Ethical approval

Approval is not necessary for a case report in our locality.

## Consent

Written informed consent was obtained from the patient for publication of this case report and accompanying images. A copy of the written consent is available for review by the Editor-in-Chief of this journal on request.

## Author contribution

Pham Thi Viet Dung: first and corresponding author, performed the operation, conceptualization, writing and revising the manuscript.

Ta Thi Hong Thuy: performed the operation, writing and revising the manuscript.

Vu Hong Chien: performed the operation, and revising the manuscript.

Truong The Duy: performed the operation, writing and revising the manuscript.

Le Anh Huy: writing and revising the manuscript.

Tran Thiet Son: reviewing and editing the manuscript.

## Registration of research studies

N/A.

## Guarantor

Tran Thiet Son. Ph.D. M.D.

## Provenance and peer review

Not commissioned, externally peer-reviewed.

## Declaration of competing interest

Authors do not report any conflict of interest.
